# A randomised controlled trial of Lichtenstein repair with Desarda repair in the management of inguinal hernias

**DOI:** 10.1016/j.amsu.2021.102486

**Published:** 2021-06-12

**Authors:** Sudhir Kumar Jain, Sushant Bhatia, Tariq Hameed, Rehan Khan, Amrita Dua

**Affiliations:** a Director-Professor, Department of General Surgery, Maulana Azad Medical College and Lok Nayak Hospital, New Delhi, India; b MCh. Resident, Department of GI Surgery & Liver Transplant, All India Institute of Medical Sciences, New Delhi, India; c Assistant Professor, Department of Surgery, Maulana Azad Medical College and Lok Nayak Hospital, New Delhi, India; d Specialty Doctor Surgery, Pilgrim Hospital, Boston, United Kingdom; e Resident, Department of General Surgery, Maulana Azad Medical College and Lok Nayak Hospital, New Delhi, India

**Keywords:** Inguinal hernia, Lichtenstein, Desarda, Mesh free tension free repair, Chronic inguinodynia, Recurrence

## Abstract

**Background:**

Ever since the advent of mesh hernioplasty with low recurrence rates, surgeons have turned a blind eye towards its devastating mesh related complications. Consequently, the quest for the best hernia surgery, that is as effective as the mesh repair but lacks its complications, continues.

**Objectives:**

The present study was carried out to compare the results of the Lichtenstein repair with the Desarda repair in the treatment of inguinal hernias.

**Methods:**

A total of 77 patients with 87 hernias were randomly allocated into two groups to undergo either the Desarda repair (Group I, 39 patients with 45 hernias) or the Lichtenstein repair (Group II, Control, 38 patients with 42 hernias). 3 patients didn't complete the follow-up and were excluded from analysis. Finally, 40 hernias were analyzed in the Lichtenstein group and 44 in the Desarda group.

**Results:**

After a 6-month follow-up period it was found that neither of the two groups had any recurrence. The incidence of chronic inguinodynia was much higher in the Lichtenstein group as compared to Desarda group. The pain scores, mean operating time, mean time to return to work and analgesic requirement was much lower with the Desarda repair as compared to Lichtenstein repair.

**Conclusion:**

Desarda repair was found to be as effective as the Lichtenstein repair in terms of recurrence and better in terms of chronic inguinodynia, complications and post operative pain scores. Desarda repair requires a significantly shorter operating time. The economic burden of this repair is much less compared to mesh repair.

## Highlights

•Inguinal hernia remains one of the most commonly performed surgery by general surgeons.
•There are various techniques but researchers have focused on one with zero recurrence, less complications and less post operative pain.
•Desarda (tissue repair) is a technique where mesh is not used. It is very promising for countries with low healthcare budget like India.
•In our study Desarda has been found to have same rate of recurrence as Lichtenstein with less post operative pain (inguinodynia) and infections.
•Operating time was also less in Desarda repair, along with less requirement of post operative analgesics and early return to routine activities.

## 1 Background

Risk of getting a hernia in one's lifetime ranges from 27% in men to 3% in women [1]. Inguinal hernia is one of the most common clinical conditions encountered in the general surgical practice. Over 20 million groin hernia surgeries are carried out worldwide [2]. Before mesh repair had its day under the sun, Bassini and Shouldice repairs were the most popular. Shouldice repair relied heavily on the strength of fascia transversalis, however the recent studies have revealed that strength of posterior wall of inguinal canal lies in aponeurotic extensions from the transversus abdominis muscle and not in fascia transversalis [3]. The main objection with the above mentioned repairs was tension on the suture line which violates the most basic principle of surgery [4]. Recently mesh prosthesis have been introduced in hernia surgery but the apparent advantages of simplicity of the procedure, low recurrence rates and a relatively comfortable perioperative period have not in any manner suppressed their morbid mesh related complications [4,5].

Mesh induces fibrosis which results in stiffness and foreign body sensation, which frequently become a source of agony for the patient [6]. Mesh repair has been implicated in the rise of incidence of chronic groin pain from 1% to 28.7% [7]. The implanted mesh has been recovered from the intestine, urinary bladder, femoral vein, preperitoneal space and the scrotum revealing the true dangers of mesh usage [5]. Mesh infection is a devastating complication and may require removal of the entire mesh. The collateral damage done in this process should call all the surgeons to question the use of mesh. The mesh is not available in every part of the world and increases the cost of the procedure manifolds [8].

Desarda technique requires no complicated dissection or suturing, no mesh is needed and is easy to learn with almost zero recurrence rates [3,4,9]. This repair is based on the basic principle of providing a strong and dynamic posterior wall to the inguinal canal. A thin strip of external oblique aponeurosis reinforces the absent conjoint aponeurotic extensions to the posterior wall of the inguinal canal [10]. The ageing process is minimal in the tendons and aponeurosis thus, the use of a strip of external oblique aponeurosis, which is tendo-aponeurotic in nature in the Desarda's repair, is the best alternative to a mesh or Shouldice repair [4]. The thinned out portion of the external oblique aponeurosis which might be a concern to some surgeons doesn't affect the outcome of the repair. This repair thus eliminates all the drawbacks and complications of using a mesh [4].

The ideal surgery to treat inguinal hernia which is simple, avoids the complications of mesh implantation, has low recurrence rates and can be performed by non-consultant staff is still far from being defined. Desarda repair brings us one step closer in this regard. There is a paucity of data in the literature that compares the two procedures. The present study was carried out with an objective to compare the short term results of Lichtenstein repair versus Desarda repair in the management of inguinal hernias among adult Indian patients.

## 2 Material and methods

**Fig. 1:**
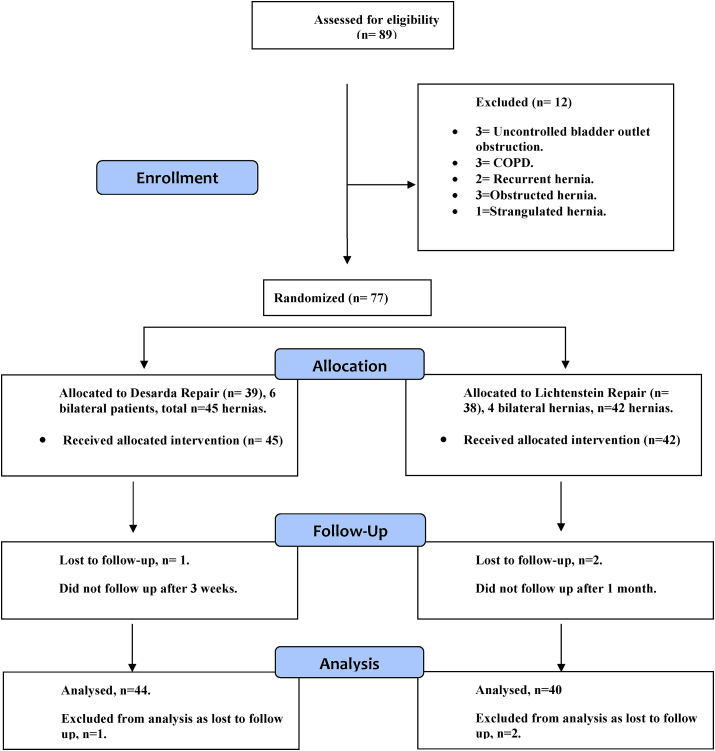
Patient flow diagram based on consort guidelines.

The present study was a randomized controlled trial conducted in the Department of General Surgery, Maulana Azad Medical College and Lok Nayak Hospital, New Delhi from December 2015 to April 2018. This research has been designed in compliance with CONSORT guidelines as studies have shown that many of the studies conducted by surgical disciples lack adherence to it [[11], [12]]. The study was registered with researchregistry.com with UIN researchregistry2789 [13]. It comprised of 77 patients having 87 inguinal hernias who presented to the surgical outdoor patient department. The study was approved by the institutional ethical committee. All the patients with reducible inguinal hernia were included in the study. The exclusion criteria were: patients under 18 year's age; patients with strangulated, recurrent, irreducible, obstructed hernias; patients with chronic cough/C.O.P.D.; patients with uncorrected bladder outlet obstruction and patients with local skin infection. Patients were divided into two groups by computer generated random numbers which were kept in a sealed envelope. The two groups were the following: one that underwent tissue based Desarda repair (group A) and the other that underwent mesh based Lichtenstein repair (group B). (Fig. 1).

**Graph 1:**
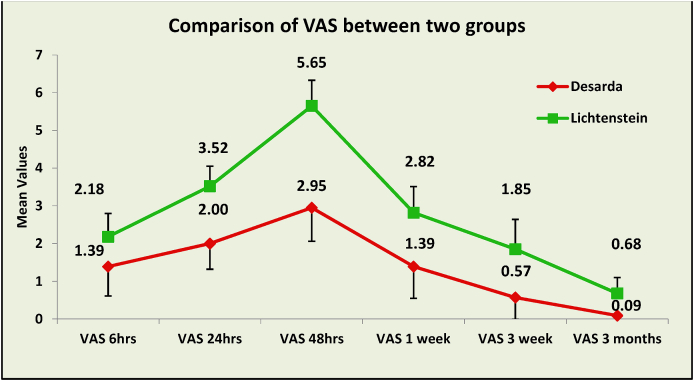
Graph depicting pain trends post-operatively.

All patients were operated as elective cases under spinal anesthesia. A prophylactic dose of Amoxicillin and Clavulanic acid (1.2 g) was given 30 min before the skin incision. The surgical site was prepared using povidone iodine (7.5%) scrub. In case of bilateral hernias, each side was assessed independently in terms of operating time, chronic inguinodynia, complications and short term recurrence.

### 2.1 Lichtenstein repair (group A)

The repair of the hernia was made with an onlay piece of mesh buttressing the floor of the inguinal canal. A polypropylene mesh of at least 15 × 10 cm in size was used. The mesh was sutured at least 2 cm beyond the pubic tubercle medially. A continuous non absorbable monofilament suture was used to suture the inferior edge of the mesh to the shelving edge of the inguinal ligament to a point 2 cm lateral to the internal ring. A slit was fashioned in the mesh to allow the cord structures to pass. Interrupted sutures were placed to fix the mesh superiorly, taking care not to include any nerves in these sutures. The tails of the mesh were placed smoothly under the external oblique laterally, frequently extending nearly to the anterior superior iliac spine. The external oblique aponeurosis was closed over the cord structures using continuous Polypropylene 2/0 suture and the skin was sutured using interrupted Nylon 3/0 sutures.

### 2.2 Desarda repair (group B)

Inguinal canal was opened through the groin incision. The thin, flimsy fascial layer over the EOA was kept intact as far as possible. This step gives additional strength to the posterior wall of inguinal canal. The superior leaf of the external oblique aponeurosis was sutured with the shelving, inturn edge of the inguinal ligament using continuous number 1 monofilament Polydioxanone suture. The first two sutures were taken through the anterior rectus sheath. The last suture was taken so as to narrow the deep ring adequately without constriction. Using a splitting incision that extended 1–2 cm beyond the deep ring laterally and medially till the pubic symphysis, a 2 cm strip of external oblique aponeurosis was fashioned out from the sutured superior leaf. This gave us a strip of external oblique aponeurosis whose lower border was already sutured to the inguinal ligament. The medial and lateral attachments of the strip were intact. The upper free border of the strip was sutured to the conjoint muscle using interrupted number 1 monofilament Polydioxanone suture. This resulted in a strip of EOA placed behind the cord to form a new posterior wall. The cord was placed in the canal and the inferior leaf of EOA was sutured to the newly formed superior leaf in front of the cord using continuous Polypropylene 2/0 suture. Skin was sutured using interrupted Nylon 3/0 sutures.

#### 2.2.1 Post-operative protocol

• All patients received injectable Diclofenac Sodium 75 mg just after the surgery, 8 p.m. on the day of the surgery and 6 a.m. on post-operative day (POD) 1. Any additional requirement was recorded.
• Injectable Amoxicillin and Clavulunic acid 1.2 g was given via intravenous route at 4 p.m. and 10 p.m. on the day of the surgery. No antibiotics oral or parenteral were given after that [25].
• Early ambulation for daily routine activities was encouraged on the next day of the surgery. Patients were discharged whenever they had tolerable pain on oral analgesics and could walk with ease. All patients were discharged on oral Diclofenac 75 mg to be taken twice a day for 3 days. Patients were told to write down any additional analgesic requirement that was recorded on follow up visits.
• Patients were asked to visit the O.P.D. on the 7th day, 21st day, 3 and 6 months after the surgery. 2 patients in the Lichtenstein group were lost to follow up and hence were excluded from the study.
• Short term recurrence rate was noted on the 6th month visit.
• Sutures were removed on the 7th day.

#### 2.2.2 Study variables

• Postoperative pain was measured using a ten-point visual analogue scale (VAS) ranging from 0 (no pain) to 10 (unbearable pain) at 6 h, 24 h, 1week, 3weeks and 3 months after surgery.
• Analgesic requirement above the basic set dosage in the protocol.
• Time to return to daily routine (bathing, brushing etc.) activities.
• Hospital stay.
• Time to return to work, i.e. complete return to preoperative functional status.
• Chronic inguinodynia which was defined by any pain or stiffness or foreign body sensation in the groin 3 months after the surgery.
• Short term (<6 months) recurrence. Recurrence was defined as any visible or palpable bulge at the site of hernia.
• Complications like hematoma, scrotal edema, epididmo-orchitis, surgical site infection as defined in the Centre for Disease Control (C.D.C.) guidelines.

#### 2.2.3 Data analysis

Statistical analysis was performed by the SPSS program for Windows, version 17.0 (SPSS, Chicago, Illinois). Continuous variables were presented as mean ± SD, and categorical variables are presented as absolute numbers and percentage. Data was checked for normality before statistical analysis. Normally distributed continuous variables were compared using the unpaired *t*-test, whereas the Mann-Whitney *U* test was used for those variables that were not normally distributed. Categorical variables were analyzed using either the Chi-square test or Fisher's exact test. For all statistical tests, a p value less than 0.05 was taken to indicate a statistically significant difference.

## 3 Results

Of all the patients, 77patients were eligible for the study. They had 87 hernias. A total of 87 hernia cases were operated. Two patients from the Lichtenstein group and one from the Desarda group were lost to follow up and hence excluded from the analysis. Finally, the analyzed patients were 44 (52.4%) in the Desarda group and 40 (47.6%) in the Lichtenstein group. The two groups were comparable in terms of age, gender, type of hernia and side of hernia (Table 1).

**Table 1:** Comparison of clinical parameters between the two groups.

Parameter	Desarda Group, n = 44	Lichtenstein Group, n = 40	p value
Number of cases	44 (52.4%)	40 (47.6%)	–
Mean Age^b^	48.82 ± 15.71	48.53 ± 14.78	0.930
Age Groups^a^			0.869
17–20 years	1, 2.3%	2, 5%	
21–40 years	12, 27.3%	10, 25%	
41–60 years	18, 40.9%	18, 45%	
61–80 years	13, 29.5%	10, 25%	
Total	44	40	
Gender	All males	All males	–
Side **(Right/Left)**^a^	26/18	26/14	0.578
Type of Hernia^a^	23/18, (52.3%, 40.9%)	24/16, (60%, 40%)	0.399
**(Indirect/Direct)**			
**Others**	Pantaloon 1, Sliding 2.	–	

a Chi square test used.

b Levine's test for equality of variances used.

### 3.1 Recurrence (Table 2)

There were no recurrences in either of the group at the end of the 6 month follow up period.

**Table 2:** Summary of operating time, chronic inguinodynia and recurrence.

Parameter	Desarda group, n = 44	Lichtenstein group, n = 40	p value
Mean operating time^a^	31.39 ± 3.55 min,	65.55 ± 3.87 min,	**<0.001**
**(Min-Max)**	22–40 min	58–78 min	
Chronic Inguinodynia^b^	4, 9.1%	25, 62.5%	**<0.001**
Recurrence	Nil	Nil	**--------**

a T test used.

b Fischer's exact test used.

### 3.2 Operating time (Table 2)

The mean operating time (Table 2) in the Desarda group was much shorter (31.39 ± 3.55 min) as compared to the Lichtenstein group (65.55 ± 3.87 min). The maximum operating time in the Desarda group was 40 min and minimum was 22 min. The maximum time in the Lichtenstein group was 78 min and minimum was 58 min. The p value for the difference in the operating time is < 0.001, thus this difference is statistically significant.

### 3.3 Pain scores and chronic inguinodynia (Table 2, Graph1)

The mean pain score (VAS) at 6 h was 1.39 ± 0.62 for Desarda repair as compared to 2.18 ± 0.78 for Lichtenstein repair. There was a rise in pain starting 48 h postoperatively in both the groups, mean score being 2.95 ± 0.68 in the Desarda group and 5.65 ± 0.89 in the Lichtenstein group. There was a subsequent fall of pain in both the groups, but the pain scores were higher in the Lichtenstein group throughout. Mean pain score at 1 week in the Desarda group was 1.39 ± 0.69 as compared to 2.82 ± 0.84 in the Lichtenstein group. The pain scores reached their minimum 3 months post operatively, mean score in the Desarda group being 0.09 ± 0.42 and in the Lichtenstein, 0.68 ± 1.02. The **“p”** value for the difference in mean scores at all the times was **<0.001**, thus statistically significant (Table 2). In our study any pain, foreign body sensation or stiffness in the inguinal region persisting at 3 months and beyond was taken as chronic inguinodynia. 4 patients (9.1%) in the Desarda group experienced chronic inguinodynia as compared to 25 patients (62.5%) in the Lichtenstein group (Table 2). The **p value was less than 0.001**. Out of the 4 patients in the Desarda group, 3 experienced stiffness and one experienced pain (VAS = 2). Out of the 25 patients in the Lichtenstein group, 11 patients experienced stiffness in the inguinal region, 11 patients experienced stiffness and pain in the inguinal region and 3 patients only experienced pain.

Time to return to normal activities and time to return to work was observed to gauge the impact of the surgery on the quality of life. The mean time was much shorter in the Desarda group, 3.48 ± 1.07 days as compared to 7.92 ± 1.73 days in the Lichtenstein group. The p was less than 0.001 (Table 3). The time to return to work was defined as the time taken to return to complete preoperative functional status. The mean time was 10.73 ± 3.78 days in the Desarda group as compared to 24.48 ± 9.43 in the Lichtenstein group. The p value was less than 0.001 (Table 3).

**Table 3:** Summary of time to return to normal activities, time to return to work, hospital stay and analgesic requirements.

Parameter	Desarda group, n = 44	Lichtenstein group, n = 40	p value
Time to return to normal activity^a^	3.48 ± 1.07	7.92 ± 1.73	**<0.001**
**(Mean days, Max-Min days)**	2–6	6–12	
Time to return to work^a^	10.73 ± 3.78	24.48 ± 9.43	**<0.001**
**(Mean days, Max-Min days)**	5–20	16–76	
Hospital stay^a^	2.34 ± 0.57	4.88 ± 1.67	**<0.001**
**(Mean days, Max-Min days)**	2–4	3–14	
Analgesic requirements^a^	156.82 ± 122.99 mg 0–450 mg (Diclofenac)	684.37 ± 254.87 mg 300–1350 mg (Diclofenac)	**<0.001**
**(Mean dosage, Max-Min dosage)**			

a T test used.

The mean hospitalization time in the Desarda group was 2.34 ± 0.57 days as compared to 4.88 ± 1.67 days in the Lichtenstein group (Table 3). The p value was less than 0.001.

#### 3.3.1 Additional analgesic requirement (Table 3)

All the patients in the Lichtenstein group needed additional supplementation of analgesics. 5 patients in the Desarda group (11.3%) did not need any additional analgesics. The total analgesic requirement was much lower in the Desarda group than the Lichtenstein group. The mean requirement in the Desarda group was 156.82 ± 122.99 mg as compared to 684.37 ± 254.87 mg in the Lichtenstein group (Table 3). The p value was less than 0.001.

### 3.4 Complications (Table 4)

The overall complication rate was higher in the Lichtenstein group (Table 4) The **p** value for this difference was **0.001** and hence was statistically significant. Scrotal edema was the most common complication in both the groups. The Lichtenstein group had 10 patients (25%) who had scrotal edema as compared to 3 patients (6.8%) in the Desarda group. The p value for this difference was 0.033 and hence statistically significant. The rates of other complications were also higher in the Lichtenstein group, but they never reached statistical significance. Only one patient in the Lichtenstein group developed mesh infection for which mesh removal was done.

**Table 4:** Summary of complication rates.

Parameter	Desarda group, n = 44	Lichtenstein group, n = 40	p value
Overall complication rate^a^	9/20.5%	22/55%	**0.001**
Epididmo-orchitis^a^	2/4.5%	3/7.5%	0.665
Hematoma^a^	3/6.8%	8/20%	0.106
Mesh Infection^a^	0	1/2.5%	0.476
Scrotal Edema^a^	3/6.8%	10/25%	**0.033**
SSI^a^	1/2.3%	2/5.0%	0.603

a Fischer exact test used.

## 4 Discussion

Evidence gathered from the Egyptian Papyrus of Ebers (1552 B.C.) reveals an incontrovertible fact that the surgery for hernia is as old as surgery itself [14]. This surgery has seen dramatic changes, from ligature of cord and sac along with castration being recommended by Paul of Aegina to laying down of the five sophisticated principles of hernia surgery namely-antisepsis, high ligation of the sac, tightening of the internal ring, reconstruction of the posterior inguinal floor and tension free repair [14]. The realization that tension on the suture line is the cause of pain and recurrence put Bassini's and Shouldice’ procedures back into the textbooks and paved way for prosthetic mesh repair. Its low recurrence rates have blindfolded the surgeons towards its disastrous complications ranging from scrotal edema to mesh migration and mesh infection. Desarda described the role of aponeurotic extensions from the transversus abdominis muscle to the posterior wall of the canal in protection against herniation [3].

In our study duration of the surgery was calculated from the time of the skin incision to the last skin suture. Duration of surgery was much shorter in Desarda than Lichtenstein repair. Similar results were achieved in various studies [[5], [15], [16], [17], [18]]. A comparative study between Desarda and Bassini for complicated inguinal hernia had similar outcome, where mean operating time in Desarda repair was less than Bassini [19]. The duration of surgery depends on various factors, some related to the surgery like its complexity, the anatomy encountered and some related to the surgeon. The shorter duration of the Desarda repair can be attributed to the lack of complicated dissection, simple steps of the surgery with little scope of modification and the absence of time consuming steps like fashioning the mesh according to the space available and placing it correctly in the inguinal canal [[5], [20]]. The shorter operating time confers many advantages to this technique like feasibility as a day care procedure and feasibility to be done under local anesthesia.

Pain scores are measured using the ten-point Visual Analogue Scale (VAS). In our study pain scores in Desarda group were lower at all post operative points observed. Our study results are in contrast to those reported by Youssef et al. [5], Szopinski et al. [21], Manyilirah et al. [16] and Rodriguez et al. [17] where no statistically significant difference was found between the pain scores of Desarda and Lichtenstein groups. In the trial by Szopinski et al. [21], pain scores were found to be slightly higher in the Desarda group on the 7th and 30th day after the surgery, but this was not statistically significant. In the study by Desarda and Ghosh [20], no patient had any pain for more than 15 days in the Desarda group but 4 patients had moderate pain and 15 had mild pain at the end of one month in the Lichtenstein group. In the retrospective study by Zulu et al. [18], pain was experienced by 3 patients who underwent Desarda repair (25%) as compared to 5 patients (21.7%) who underwent Lichtenstein repair. Higher pain scores in the Lichtenstein repair are due to the extensive dissection needed to create space for the mesh and foreign body reaction to the mesh. Lower pain scores in the Desarda repair confirm that it is a tension free repair.

Any pain, foreign body sensation or stiffness in the inguinal region persisting at 3 months and beyond was taken as chronic inguinodynia. Incidence of inguinodynia was found to be more in the Lichtenstein group as compared to Desarda group (p < 0.001). Incidence of inguinodynia is much less in Desarda repair because in this repair no mesh is used and the use of mesh leads to fibrosis which leads to stiffness. The studies by Youssef et al. [5], Szopinski et al. [21], Mitura et al. [15] report a higher rate of chronic inguinodynia in the Lichtenstein group compared to the Desarda group, but none reached statistical significance. In the study by Desarda and Ghosh [20], 2 patients reported moderate pain and 14 reported mild pain at the end of 6 months in the Lichtenstein group, no pain was reported in the Desarda group. Furthermore, 13 patients (6.4%) continued to have mild discomfort at the end of one year in the mesh group but no such pain was reported in the Desarda group. 3 patients in the mesh group needed surgery for chronic intense pain (1.47%). Our study clearly establishes the superiority of the Desarda repair in terms of chronic inguinodynia. With the advent of prosthetic materials, recurrence rates have dropped dramatically making chronic inguinodynia as the most important post operative issue. This syndrome is distressing to the patient, affects their quality of life significantly and above all, not understood quite well by the surgeons. Incidence rates ranging from 1.5% to 63% have been reported [[22], [23], [24]].

Somatic pain is the most common type and due to damage to the pubic tubercle during mesh suturing. Neuropathic pain is caused by the damage to the ilio-inguinal and genitofemoral nerves either due to injury during the surgery or due to incorporation into the inflammatory process caused by the prosthetic material. Desarda repair, being a pure tissue repair will not cause the above mentioned syndromes. However, damage to the nerves during the surgery remains a possible cause, but then again lack of extensive tissue dissection reduces its chance of occurring [[20], [24]].

In our study time to return to normal activities was significantly lower in the Desarda group. Normal activities comprised daily, routine, household activities like bathing, walking around the house etc. Time to return to work was defined as the time taken to return to complete preoperative functional status. Our study is consistent with the results by Youssef et al. [5], Rodriguez et al. [17], Desarda and Ghosh [20]. However the studies by Szopinski et al. [21], Manyilirah et al. [16] did not report any statistically significant difference in this regard. Our study establishes that the time to return to preoperative functional status is shorter with the Desarda repair. Lack of severe pain and discomfort in the Desarda group are the main reasons responsible for this. However, similar results were not achieved by all the studies. These differences can be attributed to the different definitions of return to normal activities, work in every study. The shorter return to preoperative functional status makes this repair an avid choice as a day care procedure for inguinal hernia.

In our study all the patients in the Lichtenstein group needed additional supplementation of analgesics. The total analgesic requirement was much lower in the Desarda group than the Lichtenstein group. Analgesic requirement as a separate parameter of comparison was not evaluated in the literature. In our study, the analgesic requirement was significantly lower in the Desarda group. Low pain scores in the Desarda group is the reason of such an observation.

In our study the patients were discharged when they could walk and had bearable pain. The mean hospitalization time in the Desarda group was less as compared to Lichtenstein group. Our results were consistent with those obtained by Mithura et al. [15], Rodriguez et al. [17], Desarda and Ghosh [20] and Zulu et al. [18]. Desarda repair is associated with shorter hospitalization time. Less pain scores, early return to normal activity are the main reasons behind this observation.

No recurrences were observed in either of the groups in our study till 6 months of follow up as also reported by Manyilirah et al. [16] and Zulu et al. [18]. Youssef et al. [5] reported one recurrence in each group, both of which were after one year, but this was not statistically significant. The recurrence in the Desarda group was at the newly constructed deep internal ring and in the Lichtenstein group near the pubic tubercle. Szopinski et al. [21] also reported two recurrences in each group but this too was not statistically significant. None of the recurrences were before one year. Rodriguez et al. [17] reported 4 recurrences (0.5%) in the Desarda group and 3 (0.4%) in the Lichtenstein group, again statistically not significant. Desarda and Ghosh [20] reported no recurrences in the Desarda group as opposed to 4 in the Lichtenstein group, p < 0.003, statistically significant. The recurrences in the Desarda group occurred at the site of newly constructed deep ring which seemed to be due to the faulty technique of using an inappropriately sized strip [[5], [21]]. In our study, we used a strip not more than 2 cm in width thus avoiding this. The other site of recurrence was the weakened posterior wall which was not addressed during the primary surgery [21]. In our study, we found similar weaknesses in the posterior wall intraoperatively. Aponeurotic extensions were absent at these sites and so we approximated them using interrupted sutures. Our study did not report any recurrence till 4 months, however, long term follow-up is needed to prove the validity of the results.

The overall complication rate was higher in the Lichtenstein group. Scrotal edema was the most common complication in both the groups. The rates of other complications were also higher in the Lichtenstein group, but they never reached statistical significance. These results were consistent with those by Youssef et al. [5], Szopinski et al. [21], Manyilirah et al. [16], Rodriguez et al. [17], Desarda and Ghosh [20] and Zulu et al. [18]. Extensive dissection that is required in mesh repair and the florid inflammatory reaction incited by the prosthetic material results in more postoperative edema and consequently more scrotal edema. Thus more morbidity is associated with Lichtenstein repair as compared to the Desarda repair.

Another parameter that deserves mention is the cost effectiveness of the Desarda repair. The financial burden of hernia repair although small as compared to the total health spending, is not insignificant [21]. In developing countries of Asia and Africa, where basic health care facilities are underdeveloped, the availability of mesh can be a problem. In these settings, a mesh free repair with efficacy similar, if not superior to the Lichtenstein repair would go a long way. The economic viability of this repair can be extrapolated to its low analgesic requirement, less time to return to preoperative functional status and shorter hospital stay. Thus one concrete advantage of the Desarda repair is its low cost and hence this repair is attracting the interest of many surgeons around the globe.

## 5 Conclusions

Desarda repair was found to be superior in terms of less operating time, less post-operative pain scores, less analgesic requirement, shorter hospital stay and early return to preoperative functional status. Incidence of inguinodynia was found to be much less in the Desarda group. Use of Desarda repair avoids mesh related complications like mesh infection, heaviness in the groin and foreign body sensation. Desarda repair is much more economical than Lichtenstein repair.

## Provenance and peer review

Provenance and peer review not commissioned, externally peer-reviewed.

## Sources of funding

There are no sources of funding. There is no institutional/government funding.

Authors will be paying the APC from their own pockets.

## Ethical approval

Research was given ethical approval by institutional ethics committee of Maulana Azad Medical College, New Delhi, India on November 27, 2015 with the reference number, F. No./11/IEC/MAMC/2015/317.

## Consent

Informed written consent was taken from all participants of the study.

## Author contribution

SKJ conceived and designed the study. SB, TH, RK, and AD contributed to drafting and revising the manuscript critically for important intellectual content. SKJ, SB, and TH supervised data collection and management. SB, TH, and AD assisted with patient recruitment and consent. SB, TH, RK and SKJ developed the statistical analysis plan for outcomes. SKJ and TH oversaw the data quality and revised the manuscript as needed. SKJ is the guarantor of this work. All of the authors approved the final version and agreed to be accountable for all aspects of the work.

## Registration of research studies

1. Name of the registry: CTRI
2. Unique Identifying number or registration ID: The trial has been noted in the national clinical trial registry of India (http://ctri.nic.in/), with the reference number REF/2017/05/014435, pending confirmation.
3. Hyperlink to your specific registration (must be publicly accessible and will be checked):

## Guarantor

Dr. Sudhir Kumar Jain.

Director Professor, Department of Surgery.

Maulana Azad Medical College and Lok Nayak Hospital.

New Delhi, India.

PIN- 110002.

## Declaration of competing interest

There are no conflicts of interests. There is no financial or personal relationship that could inappropriately influence our work.
